# Supplementary material for: Comparison of Healthiness, Labelling, and Price between Private and Branded Label Packaged Foods in New Zealand (2015–2019)
Source: Nutrients. 2021 Aug 9;13(8):2731. doi: 10.3390/nu13082731 (PMC8401692; doi:10.3390/nu13082731)
Supplement: Supplementary file 1 [file nutrients-13-02731-s001.zip › nutrients-1317391-supplementary.pdf]

Table S1. Description and number of products assessed by food category from 2015-2019 (overall and by private and branded label).

Food category	Type of products included in category	Number of products included		
		Total	PL	BL
	Bread and bakery products			
Savoury biscuits	(L3): Plain dry biscuits - (L4): Other gluten-free crackers and crispbread; Other plain dry biscuits; Rice cakes and similar products (plain); Rice crackers (plain); Wheat-based crackers (e.g. water crackers); Wheat-based crispbread (e.g. Salada).<br>(L3): Savoury biscuits - (L4): Breadsticks; Flavoured rice cakes and corn cakes; Flavoured rice crackers; Other savoury crackers; Wheat-based flavoured crackers and crispbread.	1623	276	1347
Sweet biscuits	(L3): Gluten-free sweet biscuits.<br>(L3): Sweet filled biscuits - (L4): Chocolate-covered sandwich style biscuits (e.g. Tim Tam); Fruit filled biscuits; Jam filled biscuits; Other sweet filled biscuits; Anzac style biscuits; Choc-chip biscuits; Chocolate coated biscuits.<br>(L3): Sweet unfilled biscuits - (L4): Fruit and nut chunks biscuits; Gingerbread biscuits; Iced biscuits; Oatmeal/wheatmeal biscuits; Other sweet unfilled biscuits; Plain sweet unfilled biscuits; Shortbread style biscuits.	2102	375	1727
Everyday sliced breads	(L3): Mixed grain bread - (L4): Gluten free grain breads; Mixed grain sourdough; Multigrain bread; Other grain breads; Rye bread; Soy & linseed bread.<br>(L3): White bread.<br>(L3): Wholemeal bread.	657	109	548
Other breads	(L3): Flat bread - (L4): Gluten-free flat bread; Naan; Pita bread; Regular flat bread; Roti; Tortillas; Pizza bases.<br>(L3): Other bread - (L4): Bagels; English muffins; Gluten-free bread; Other plain bread; Other savoury bread; Savoury bread rolls.	1325	259	1066
Cakes and muffins: ready-to-eat	(L3): Cakes: Bavarians; Brownies; Carrot cakes; Cheesecakes; Chocolate cakes, cupcakes; Doughnuts; Fruit cakes; Other cakes; Pancakes; Swiss rolls; Vanilla/plain cakes.<br>(L3): Muffins - (L4): Chocolate/choc-chip muffins; Fruit-based muffins; Other sweet muffins.	568	120	448
	Cereal and cereal products			
Breakfast cereals: ready-to-eat	(L3): Ready-to-eat - (L4): Brans (excludes unprocessed ones); Flakes; Granola/cluster cereals; Muesli; Puffed cereals; Sweet cereals.<br>(L3): Ready-to-eat - (L4): Flakes - (L5): Cornflakes; Flakes with additions; Other plain flakes.<br>(L3): Ready-to-eat - (L4): Muesli - (L5): Muesli with fruit; Plain muesli.<br>(L3): Ready-to-eat - (L4): Puffed cereals - (L5): Cocoa pop style cereal; Other puffed cereals; Rice bubbles.<br>(L3): Ready-to-eat - (L4): Sweet cereals - (L5): Cocoa-based; Fruit-loop style; Nutri-Grain style cereal; Other sweet style cereal.<br>(L3): Ready-to-eat - (L4) Breakfast biscuits.	1506	221	1285
Cereal bars	(L3): Cereal-based bars - (L4): Baked/filled bars; Chocolate-coated cereal-based bars; Muffin bars; Plain cereal-based bars; Snack logs; Variety packs cereal and nut bars; Yoghurt-coated cereal-based bars.	1050	190	860

	(L3): Nut-based bars - (L4): Chocolate-coated nut-based bars; Plain nut-based bars; Yoghurt-coated nut-based bars.<br>(L3): Puff-based bars.			
	Dairy			
Ice cream	(L3): Ice-cream - (L4): Vanilla ice cream; Other sweet-based ice cream; Other chocolate-based ice cream; Ice cream stick - choc coated; ice cream cups; Ice cream cones (e.g. Cornetto); ice cream cakes; Fruit-flavoured ice cream; Chocolate ice cream.	1612	140	1472
	Fish and seafood products			
Canned fish	(L3): Canned fish - (L4): Canned salmon - (L5): Plain canned salmon, Flavoured canned salmon; (L4): Canned tuna - (L5): Flavoured canned tuna, Plain canned tuna in oil, Plain canned tuna in water; (L4): Canned sardines - (L5): Plain canned sardines, Flavoured canned sardines; (L4): Other canned fish.	1100	324	776
	Fruit and vegetables			
Fruit in syrup/juice	(L3): Fruit in juice/syrup - (L4): Pineapple in juice/syrup, Pears in juice/syrup, Peaches in juice/syrup, Apricot in juice/syrup, Other fruits in juice/syrup, Mixed fruits in juice/syrup.	744	357	387
Salted nuts	(L3): salted nuts - (L4): Seeds, Pistachios, Peanuts, Mixed nuts, Cashews, Almonds.	487	154	333
Vegetables-canned	(L3): Canned vegetables - (L4): Other canned vegetables, Canned tomatoes, Canned legumes, Canned corn, Canned beetroot, Canned asparagus, Baked beans.	1306	332	974
Vegetables-pickled	(L3): Pickled vegetables - (L4): Sundried tomatoes, Pickles/gherkins, Peppers/Capsicum, Other pickled vegetables, Onions, Olives, Capers, Artichokes.	976	117	859
	Meat and meat products (processed)			
Salamis, hams, bacon	(L3): Bacon.<br>(L3): Salami and cured meats - (L4): Salami, Polish Salami, Pancetta and prosciutto, Kransky, Chorizo, Cabanossi and twiggy sticks.<br>(L3): Sliced meat (excl salami and other cured meat) - (L4): Sliced Turkey, Slice Pork, Sliced meat Variety packs, Sliced Luncheon meat, Slice ham, Sliced chicken, Sliced beef.<br>(L3): Whole hams and similar products.	1616	205	1411
Sausages, hotdogs	(L3): Sausages and hotdogs - (L4): Sausages - (L5): Beef sausages, Chicken Sausages, Lamb sausages, Pork sausages, Other sausages. (L4): Hotdogs, Cocktail sausages.	760	153	607
Raw or frozen meats with flavour/coated	(L3): Frozen meat - (L4): Coated frozen meat.<br>(L3): Kebabs.<br>(L3): Meat burgers - (L4): Other meat burgers, Lamb meat burgers, Chicken burgers, beef burgers.<br>(L3): Other meat products (includes different types/varieties of seasoned/coated meats).<br>(L3): Raw flavoured meats - (L4): Raw flavoured turkey, Raw flavoured pork, Raw flavoured lamb, Raw flavoured chicken, Raw flavoured beef.	972	177	795
	Sauces and spreads			
Mayonnaise and salad dressings	(L3): Mayonnaise.<br>(L3): Dressings - (L4): Other, French, Italian, Creamy, Balsamics.	970	127	843
Pasta sauces	(L3): Pasta sauces - (L4): Pesto; (L4): Fresh pasta sauces - (L5): Cream-based fresh pasta sauces, Tomato-based fresh pasta sauces; (L4): Ambient pasta sauces - (L5): Cream-based ambient pasta sauces, Pasta sauce with meat, Tomato-based ambient pasta sauces.	926	150	776

Savoury spreads and dips	(L3): Savoury spreads - (L4): Relishes and pickles - (L5): Chutney, Pickles, Relishes; (L4): Other savoury spreads.<br>(L3): Dips - (L4): Salsa, Chilled dips.	1601	171	1430
Peanut butter and other nut-based spreads	(L3): Peanut butter - (L4): Smooth peanut butter, Crunchy peanut butter.<br>(L3): Other nut-based spreads.	442	109	333
	Snack foods			
Crisps and salty snacks	(L3): Salt and vinegar.<br>(L3): Pretzels.<br>(L3): Gluten-free corn chips.<br>(L3): Extruded snacks.<br>(L3): Corn chips.<br>(L3): Potato Crisps - (L4): Sweet chilli, Sour cream and chives, Plain, Other flavours, Chicken flavour, Cheese and onion, BBQ flavour.	1241	200	1041
	All products			
All products		23,584	4,266	19,318

L3: Nutritrack level 3; L4: Nutritrack level 4; L5: Nutritrack level 5; PL: private labels; BL: branded labels.

Table S2. Number and proportion of products with missing information for sugar content, sodium content, estimated HSR and HSR displayed on front-of-pack-labelling from 2015-2019, by food category (overall).

Food categories	Number of products in category	Missing information - N (%)			
		Sugar (g/100 g)	Sodium (mg/100g)	Estimated HSR	HSR displayed on front-of-pack
Bread and bakery products					
Savoury biscuits	1623	7 (0.4)	14 (0.8)	19 (1.2)	0 (0.0)
Sweet biscuits	2102	61 (2.9)	66 (3.1)	101 (4.8)	0 (0.0)
Everyday sliced breads	657	14 (2.1)	18 (2.7)	20 (3.0)	0 (0.0)
Other breads	1325	11 (0.8)	33 (2.5)	38 (2.9)	0 (0.0)
Cakes and muffins: ready-to-eat	568	16 (2.8)	19 (3.3)	28 (4.9)	0 (0.0)
Cereal and cereal products					
Breakfast cereals: ready-to-eat	1506	10 (0.7)	12 (0.8)	19 (1.3)	0 (0.0)
Cereal bars	1050	7 (0.5)	7 (0.5)	13 (0.9)	0 (0.0)
Dairy					
Ice-cream	1612	9 (0.6)	--	17 (1.1)	0 (0.0)
Fish and seafood products					
Canned fish	1100	--	27 (2.5)	38 (3.5)	0 (0.0)
Fruit and vegetables					
Fruit in syrup/juice	744	3 (0.4)	--	13 (1.7)	0 (0.0)
Salted nuts	487	--	9 (1.8)	13 (2.7)	0 (0.0)
Vegetables - canned	1306	--	19 (1.5)	37 (2.8)	0 (0.0)
Vegetables - pickled	976	--	25 (2.6)	67 (6.9)	0 (0.0)
Meat and meat products (processed)					
Salamis, hams, bacon	1616	--	17 (1.1)	26 (1.6)	0 (0.0)
Sausages, hotdogs	760	--	11 (1.4)	18 (2.4)	0 (0.0)
Raw or frozen meats with flavour/coated	972	--	9 (0.9)	15 (1.5)	0 (0.0)
Sauces and spreads					
Mayonnaise and salad dressings	970	31 (3.2)	14 (1.4)	56 (5.8)	0 (0.0)
Pasta sauces	926	16 (1.7)	11 (1.2)	19 (2.1)	0 (0.0)
Savoury spreads and dips	1601	18 (1.1)	16 (1.0)	27 (1.7)	0 (0.0)
Peanut butter and other nut-based spreads	442	4 (0.9)	1 (0.2)	4 (0.9)	0 (0.0)
Snack foods					
Crisps and salty snacks	1241	--	9 (0.7)	18 (1.5)	0 (0.0)

HSR: Health Star Rating.

--Nutrient content not assessed as food category does not represent relevant source of the nutrient.

Table S3. Sodium content within the selected food categories: mean (SD) from 2015-2019- in total, for branded and private labels.

Food categories	Sodium (mg/100 g)													
	2015		2016		2017		2018		2019		Total-2015-2019			
	n	Mean (SD)	n	Mean (SD)	n	Mean (SD)	n	Mean (SD)	n	Mean (SD)	n	Mean (SD)	Min	Max
**Savoury biscuits**														
Total	307	678.5 (346.8)	301	637.2 (304.4)	313	647.0 (296.1)	344	640.1 (272.3)	345	616.1 (256.0)	1609	642.6 (295.1)	1.0	1770.0
Branded labels	259	707.6 (355.0)	248	660.8 (308.4)	252	660.1 (298.4)	281	647.4 (274.9)	295	624.6 (263.7)	1334	658.4 (300.6)	1.0	1770.0
Private labels	48	521.9 (247.9)	53	526.6 (260.3)	61	593.0 (282.3)	63	607.4 (260.3)	50	565.7 (200.1)	275	566.1 (254.0)	100.0	1290.0
**Sweet biscuits**														
Total	390	259.9 (134.8)	404	258.2 (131.7)	377	280.0 (150.7)	436	300.0 (477.2)	429	282.1 (137.1)	2036	276.5 (253.0)	0.2	9800.0
Branded labels	310	273.9 (133.2)	316	268.9 (131.4)	312	287.7 (147.9)	374	307.6 (511.7)	356	286.4 (135.7)	1668	285.8 (270.8)	0.2	9800.0
Private labels	80	205.5 (127.6)	88	219.9 (126.1)	65	240.5 (159.3)	62	253.8 (143.4)	73	261.2 (143.1)	368	234.3 (139.9)	14.0	835.0
**Everyday sliced breads**														
Total	144	421.7 (70.4)	134	416.0 (63.0)	122	405.5 (51.8)	125	415.0 (54.5)	114	396.3 (70.9)	639	411.6 (63.2)	38.0	823.0
Branded labels	112	417.0 (75.7)	104	409.6 (66.6)	107	406.7 (53.3)	109	416.2 (56.3)	105	398.3 (73.3)	537	409.7 (65.8)	38.0	823.0
Private labels	32	438.1 (44.3)	30	438.3 (42.2)	15	397.2 (39.6)	16	406.3 (40.6)	9	373.3 (22.9)	102	421.4 (46.0)	343.0	542.7
**Other breads**														
Total	260	446.7 (172.2)	280	449.6 (160.3)	244	445.6 (156.6)	251	429.7 (143.8)	254	415.3 (145.5)	1289	437.6 (156.5)	0.0	1380.0
Branded labels	195	438.2 (189.3)	204	439.5 (169.7)	197	442.8 (162.7)	214	432.0 (142.2)	223	421.0 (144.9)	1033	434.3 (161.8)	0.0	1380.0
Private labels	65	472.1 (102.7)	76	476.6 (129.0)	47	456.0 (129.4)	37	417.0 (154.2)	31	375.0 (146.0)	256	450.7 (132.7)	15.0	824.0
**Cakes/Muffins: ready-to-eat**														
Total	112	314.7 (143.0)	125	280.7 (146.9)	94	268.9 (135.2)	105	288.5 (131.3)	113	279.2 (145.3)	546	286.8 (141.6)	24.0	763.0
Branded labels	71	297.4 (144.3)	93	273.6 (143.3)	84	266.4 (128.6)	91	288.9 (129.1)	98	288.9 (138.9)	434	282.6 (137.1)	24.0	763.0
Private labels	41	344.6 (137.0)	32	301.1 (157.7)	10	289.9 (189.4)	14	286.1 (150.1)	15	216.2 (174.1)	112	302.8 (157.4)	81.0	684.0
**Breakfast cereals: ready-to-eat**														
Total	262	203.6 (176.7)	313	180.2 (168.9)	293	177.5 (171.1)	293	167.3 (160.6)	321	167.2 (158.2)	1482	178.4 (167.1)	0.0	710.0
Branded labels	218	198.5 (172.8)	260	168.7 (159.7)	247	172.0 (163.4)	258	166.2 (158.3)	280	165.9 (155.1)	1263	173.4 (161.6)	0.0	710.0
Private labels	44	228.8 (194.9)	53	236.8 (200.4)	46	206.7 (207.2)	35	175.5 (178.8)	41	176.5 (180.3)	219	207.7 (193.7)	2.0	695.0
**Cereal bars**														
Total	188	165.5 (104.4)	229	142.4 (112.0)	221	135.2 (103.7)	215	131.5 (101.8)	190	147.6 (104.4)	1043	143.8 (105.9)	3.0	670.0
Branded labels	153	165.8 (106.0)	184	144.9 (116.8)	181	136.7 (106.2)	180	131.5 (103.7)	156	152.0 (106.0)	854	145.4 (108.4)	3.0	670.0
Private labels	35	164.2 (98.3)	45	132.3 (90.1)	40	128.8 (92.6)	35	131.5 (92.4)	34	127.3 (95.5)	189	136.4 (93.6)	16.0	351.0
**Canned fish**														
Total	242	394.4 (129.2)	220	385.9 (145.4)	226	397.6 (126.3)	195	412.7 (152.1)	190	403.6 (148.1)	1073	398.3 (139.8)	0.1	1668.0
Branded labels	167	402.1 (136.6)	140	403.1 (157.9)	149	416.9 (132.3)	145	429.0 (163.8)	149	423.5 (153.8)	750	414.7 (148.9)	0.1	1668.0
Private labels	75	377.5 (109.7)	80	355.8 (115.4)	77	360.3 (104.7)	50	365.3 (98.4)	41	331.3 (96.0)	323	360.3 (106.9)	59.0	600.0
**Nuts-salted**														
Total	75	389.7 (242.5)	104	400.6 (232.6)	94	397.2 (226.0)	101	374.8 (238.8)	104	434.6 (293.5)	478	400.1 (248.5)	3.0	1470.0
Branded labels	47	390.1 (258.1)	73	395.6 (231.7)	65	397.5 (236.2)	68	381.5 (264.2)	73	459.6 (324.2)	326	406.6 (266.1)	3.0	1470.0
Private labels	28	389.1 (218.4)	31	412.3 (238.3)	29	396.3 (205.3)	33	360.9 (178.2)	31	375.6 (195.7)	152	386.3 (205.6)	12.0	1170.0
**Vegetables-canned**														
Total	255	216.2 (182.1)	271	252.9 (346.2)	277	220.2 (163.9)	243	231.4 (203.8)	241	200.3 (155.2)	1287	224.7 (223.8)	0.0	3700.0
Branded labels	186	233.6 (194.2)	196	266.5 (309.3)	201	240.4 (170.0)	191	244.3 (217.4)	181	221.9 (161.2)	955	241.7 (217.8)	0.0	3400.0
Private labels	69	169.2 (134.6)	75	217.2 (428.2)	76	166.7 (133.6)	52	183.9 (134.8)	60	135.3 (114.0)	332	175.7 (233.7)	1.0	3700.0
**Vegetables-pickled**														

Total	156	1197.4 (1204.0)	174	1163.6 (1082.2)	197	998.8 (626.7)	218	1086.8 (943.2)	206	1056.1 (964.9)	951	1094.1 (969.4)	5.0	12000.0
Branded labels	137	1228.0 (1272.6)	153	1195.9 (1137.7)	181	100.7 (639.1)	191	1086.8 (992.6)	177	1057.9 (1023.2)	839	1105.1 (1017.6)	5.0	12000.0
Private labels	19	976.9 (433.5)	21	928.6 (477.5)	16	978.1 (479.5)	27	1086.4 (473.6)	**29**	1044.9 (484.8)	112	1012.0 (466.7)	95.0	1780.0
**Salamis, hams,bacon**														
Total	329	1257.5 (537.0)	306	1237.1 (400.8)	320	1212.2 (490.1)	337	1181.2 (373.1)	307	1201.7 (431.8)	1599	1217.7 (451.1)	212.0	6850.0
Branded labels	290	1273.3 (554.7)	265	1248.4 (410.0)	280	1217.1 (510.2)	296	1183.2 (378.0)	263	1204.3 (438.8)	1394	1225.1 (464.1)	212.0	6850.0
Private labels	39	1140.3 (363.4)	41	1163.7 (330.0)	40	1177.4 (317.3)	41	1166.5 (339.2)	44	1185.9 (392.1)	205	1167.2 (347.0)	456.0	2830.0
**Sausages, hotdogs**														
Total	148	750.1 (171.3)	183	751.2 (178.4)	161	757.5 (168.5)	135	753.8 (152.5)	122	740.4 (183.3)	749	751.0 (171.0)	280.0	1667.0
Branded labels	116	762.7 (174.6)	137	775.2 (174.7)	137	772.9 (169.8)	108	771.4 (155.7)	102	764.8 (187.0)	600	769.6 (172.0)	303.0	1667.0
Private labels	32	704.6 (152.7)	46	679.5 (171.9)	24	674.6 (136.1)	27	683.3 (116.7)	20	616.0 (93.0)	149	676.3 (144.7)	280.0	1100.0
**Raw of frozen meats with flavour/coated**														
Total	173	499.9 (225.9)	208	494.5 (222.3)	185	525.6 (206.7)	206	507.7 (211.1)	191	486.5 (203.8)	963	502.7 (214.0)	41.0	1620.0
Branded labels	132	527.8 (230.8)	169	518.1 (225.6)	161	531.0 (210.3)	172	511.2 (218.2)	153	495.3 (204.3)	787	516.4 (217.6)	41.0	1620.0
Private labels	41	410.0 (184.5)	39	392 .2 (175.9)	24	489.0 (180.1)	34	490.1 (172.5)	38	451.1 (200.4)	176	441.2 (185.6)	51.0	900.0
**Mayonnaise and salad dressings**	179	715.1 (418.7)	181	710.2 (377.4)	202	686.8 (412.2)	204	701.1 (418.2)	190	687.6 (426.4)	956	699.7 (410.6)	0.0	3000.0
Total	157	710.1 (440.3)	158	700.8 (388.9)	181	685.9 (430.7)	178	709.3 (441.7)	156	686.9 (452.4)	830	698.5 (430.6)	0.0	3000.0
Branded labels	22	750.7 (210.2)	23	774.7 (284.7)	21	694.3 (196.7)	26	645.0 (187.3)	34	690.6 (282.5)	126	707.7 (240.9)	274.0	1840.0
Private labels														
**Pasta sauces**														
Total	171	406.0 (208.4)	181	415.1 (224.1)	195	421.5 (247.8)	173	1137.0 (816.8)	195	400.7 (232.2)	915	408.8 (228.6)	5.2	1600.0
Branded labels	150	409.1 (214.5)	151	416.0 (231.0)	168	428.9 (259.6)	142	400.6 (233.1)	156	407.9 (242.7)	767	413.0 (236.9)	5.2	1600.0
Private labels	21	384.0 (160.9)	30	410.3 (188.8)	27	375.6 (150.3)	31	397.2 (203.0)	39	371.9 (184.1)	148	387.4 (178.7)	40.0	1070.0
**Savoury spreads and dips**														
Total	276	521.8 (336.4)	311	506.7 (397.8)	311	519.3 (424.4)	350	492.2 (341.4)	337	489.0 (331.6)	1585	504.8 (367.5)	0.1	4300.0
Branded labels	237	531.2 (357.3)	271	513.1 (422.7)	282	525.0 (442.2)	319	493.4 (354.7)	305	492.7 (344.8)	1414	509.7 (385.4)	0.1	4300.0
Private labels	39	465.0 (149.1)	40	463.1 (135.4)	29	463.9 (168.1)	31	479.2 (150.0)	32	453.6 (157.6)	171	464.8 (149.6)	58.4	780.0
**Peanut butter and other nut-based spreads**														
Total	64	229.0 (178.3)	88	202.3 (172.6)	85	187.2 (163.1)	99	178.0 (160.7)	105	174.4 (154.5)	441	191.1 (165.1)	0.0	955.0
Branded labels	48	213.7 (186.2)	64	179.7 (161.2)	63	176.4 (161.0)	77	161.9 (157.5)	80	163.8 (151.9)	332	176.0 (161.9)	0.0	955.0
Private labels	16	275.1 (147.7)	24	262.4 (190.6)	22	218.0 (168.8)	22	234.0 (162.9)	25	208.4 (160.7)	109	237.2 (166.8)	5.0	500.0
**Crisps and salty snacks**														
Total	226	674.0 (305.1)	250	664.1 (332.8)	253	642.5 (348.1)	252	590.4 (297.0)	251	624.5 (357.2)	1232	638.4 (330.1)	0.0	3000.0
Branded labels	186	663.2 (299.4)	203	651.9 (331.9)	210	639.9 (351.8)	217	579.5 (287.2)	216	625.1 (365.4)	1032	630.7 (329.8)	0.0	3000.0
Private labels	40	724.1 (329.4)	47	717.0 (335.2)	43	655.1 (332.7)	35	658.0 (347.7)	35	621.2 (306.5)	200	678.0 (330.0)	175.0	1700.0

SD: Standard deviation; Min: minimum; Max: maximum. Number of missing for sodium (n, 2015-19): Savoury biscuits (13); Sweet biscuits (66); Everyday sliced breads (18); Other breads (33); Cakes/Muffins: ready-to-eat (19); Breakfast cereals: ready-to-eat (12); Cereal bars (7); Canned fish (27); Nuts-salted (9); Vegetables-canned (19); Vegetables-pickled (25); Processed meats-I (17); Processed meats-II (11); Processed meats-III (9); Mayonnaise and salad dressings (14); Pasta sauces (11); Spreads-I-Savoury (16); Spreads-II-peanut butter and other nut-based spreads (1); Crisps and snacks (9).

Table S4. Sugar content within the selected food categories: mean (SD) from 2015-2019- in total, for branded and private labels.

Food categories	Sugar (g//100 g)													
	2015		2016		2017		2018		2019		Total-2015-2019			
	n	Mean (SD)	n	Mean (SD)	n	Mean (SD)	n	Mean (SD)	n	Mean (SD)	n	Mean (SD)	Min	Max
**Savoury biscuits**														
Total	308	3.0(3.2)	304	2.9(3.3)	315	3.0(3.5)	344	3.4(4.2)	345	3.4(4.1)	1616	3.2(3.7)	0.0	47.0
Branded labels	260	3.0(2.8)	251	2.9(3.0)	254	2.9(3.1)	281	3.4(4.1)	295	3.4(4.3)	1341	3.1(3.5)	0.0	25.0
Private labels	48	3.5(4.9)	53	3.1(4.7)	61	3.3(4.8)	63	3.8(4.5)	50	3.0(2.5)	275	3.4(4.4)	0.0	32.4
**Sweet biscuits**														
Total	396	32.6(10.3)	410	32.6(9.5)	373	33.3(9.9)	433	33.1(11.1)	429	32.9(10.9)	2041	32.9(10.4)	0.5	77.0
Branded labels	315	32.4(10.5)	322	32.7(9.7)	308	33.5(9.9)	371	33.1(11.4)	356	32.9(11.2)	1672	33.0(10.6)	0.5	77.0
Private labels	81	33.8(9.3)	88	32.3(9.1)	65	32.7(10.1)	62	33.2(9.0)	73	33.1(9.6)	369	33.0(9.4)	3.0	63.0
**Everyday sliced breads**														
Total	147	2.7(1.1)	134	2.7(1.1)	122	2.8(1.0)	126	3.0(1.1)	114	2.8(1.0)	643	2.8(1.1)	0.0	7.8
Branded labels	115	2.8(1.1)	104	2.8(1.0)	107	2.7(1.1)	110	2.9(1.1)	105	2.8(1.0)	541	2.8(1.1)	0.0	7.8
Private labels	32	2.3(1.2)	30	2.2(1.2)	15	3.3(0.5)	16	3.3(0.5)	9	3.2(0.8)	102	2.7(1.1)	0.3	4.6
**Other breads**														
Total	265	3.1(3.3)	287	3.2(3.8)	248	.2(3.4)	255	2.9(2.5)	256	2.8(2.0)	1311	3.0(3.1)	0.0	43.2
Branded labels	200	3.1(2.6)	211	3.3(3.9)	201	3.3(3.6)	218	3.0(2.7)	225	2.8(2.0)	1055	3.1(3.0)	0.0	43.2
Private labels	65	3.2(4.8)	76	2.9(3.4)	47	2.7(2.1)	37	2.6(1.7)	31	2.6(1.5)	256	2.9(3.3)	0.1	27.4
**Cakes/Muffins: ready-to-eat**														
Total	115	34.0(13.6)	123	32.9(13.4)	94	31.9(13.1)	104	32.1(13.2)	113	30.7(12.5)	549	32.3(13.1)	3.0	76.7
Branded labels	74	32.0(13.3)	93	32.2(13.9)	84	31.3(12.6)	90	31.2(12.7)	98	30.5(12.3)	439	31.4(12.9)	4.2	76.7
Private labels	41	36.3(14.0)	30	34.9(11.5)	10	36.6(16.4)	14	38.1(14.9)	15	31.6(13.9)	110	35.5(13.6)	3.0	74.5
**Breakfast cereals: ready-to-eat**														
Total	265	18.8(8.9)	317	18.0(8.6)	293	17.3(8.7)	293	17.1(8.1)	316	17.2(8.1)	1484	17.7(8.5)	0.0	54.6
Branded labels	220	18.5(8.2)	263	17.7(8.2)	247	16.9(8.1)	258	17.0(8.0)	275	17.2(8.1)	1263	17.4(8.1)	0.0	41.3
Private labels	45	20.3(11.8)	54	19.5(10.6)	46	19.8(11.0)	35	18.1(9.1)	41	17.6(8.3)	221	19.1(10.3)	2.0	54.6
**Cereal bars**														
Total	188	27.7(7.0)	229	26.5(7.0)	221	26.2(7.8)	215	25.6(8.1)	190	25.7(9.1)	1043	26.3(7.8)	1.5	76.1
Branded labels	153	28.1(6.8)	184	26.2(7.3)	181	26.0(8.2)	180	25.3(8.5)	156	25.4(9.7)	854	26.2(8.2)	1.5	76.1
Private labels	35	25.8(7.7)	45	27.4(5.3)	40	27.0(5.7)	35	27.1(5.0)	34	27.0(4.9)	189	26.9(5.8)	6.8	40.7
**Ice-cream**														
Total	272	23.4(4.8)	318	23.4(4.7)	308	22.6(4.7)	340	22.4(5.1)	365	22.0(5.9)	1603	22.7(5.1)	1.0	53.6
Branded labels	248	23.5(4.9)	288	23.5(4.8)	274	22.6(4.9)	313	22.3(5.3)	340	22.0(6.1)	1463	22.7(5.3)	1.0	53.6
Private labels	24	22.8(3.1)	30	22.8(2.6)	34	22.7(2.8)	27	23.4(3.1)	25	23.1(3.3)	140	23.0(2.9)	18.9	30.3
**Fruit- canned in syrup/juice**														
Total	167	13.4(5.4)	151	14.1(5.4)	151	13.9(5.4)	134	13.5(6.1)	138	12.6(4.7)	741	13.5(5.4)	0.2	53.9
Branded labels	95	13.196.3)	78	14.0(6.7)	75	14.2(6.4)	67	14.1(7.4)	71	13.4(5.5)	386	13.7(6.5)	0.2	53.9
Private labels	72	13.693.7)	73	14.2(3.7)	76	13.6(4.0)	67	13.0(4.5)	67	11.7(3.7)	355	13.3(4.0	1.6	22.3
**Mayonnaise and salad dressings**														
Total	175	12.2(13.1)	177	10.6(11.0)	198	12.5(13.3)	201	11.4(11.8)	188	11.4(12.0)	939	11.6(12.3)	0.0	90.0
Branded labels	153	12.6(13.7)	154	10.5(11.5)	177	12.7(13.8)	175	11.8(12.5)	154	11.8(12.9)	813	11.9(12.9)	0.0	90.0
Private labels	22	9.8(6.7)	23	11.5(6.9)	21	10.4(7.0)	26	9.1(5.7)	34	10.0(6.4)	126	10.1(6.5)	0.0	26.6
**Pasta sauces**														

Total	172	4.3(2.2)	181	4.4(2.1)	195	4.4(2.2)	172	4.6(2.2)	190	4.4(2.1)	910	4.4(2.2)	0.0	12.0
Branded labels	150	4.2(2.2)	151	4.4(2.1)	168	4.3(2.1)	141	4.5(2.2)	151	4.4(2.1)	761	4.3(2.1)	0.0	12.0
Private labels	22	4.9(2.3)	30	4.9(2.2)	27	5.0(2.5)	31	5.0(2.5)	39	4.5(2.1)	149	4.8(2.3)	1.0	8.8
**Savoury spread and dips**														
Total	276	12.9(14.3)	312	15.5(15.8)	311	14.0(13.9)	350	13.4(13.7)	334	12.0(13.8)	1583	13.5(14.3)	0.0	81.0
Branded labels	238	12.9(14.8)	272	15.5(16.2)	282	14.0(14.3)	319	13.5(14.1)	302	12.3(14.2)	1413	13.6(14.7)	0.0	81.0
Private labels	38	13.2(10.6)	40	15.2(12.5)	29	13.8(9.8)	31	12.7(9.7)	32	9.0(9.0)	170	12.9(10.6)	0.5	54.5
**Peanut butter and other nut-based spreads**														
Total	63	6.7(7.1)	88	7.7(9.8)	84	5.3(2.8)	98	5.6(3.2)	105	5.8(3.1)	438	6.2(5.8)	0.5	60.0
Branded labels	47	5.4(2.6)	64	6.6(7.5)	62	5.2(2.5)	76	5.5(3.1)	80	5.9(3.1)	329	5.7(4.2)	0.5	60.0
Private labels	16	10.6(13.0)	24	10.8(14.0)	22	5.8(3.4)	22	6.0(3.5)	25	5.6(3.1)	109	7.6(8.8)	1.0	56.8

SD: Standard deviation; Min: minimum; Max: maximum. Number of missing for sugar (2015-19): Savoury biscuits (6); Sweet biscuits (61); Everyday sliced breads (14); Other breads (11); Cakes/Muffins: ready-to-eat (16); Breakfast cereals: ready-to-eat (10); Cereal bars (7); Ice-cream (9); Fruit- canned in syrup/juice (3); Mayonnaise and salad dressings (31); Pasta sauces (16); Spreads-I-Savoury (18); Spreads-II-peanut butter and other nut-based spreads (4).
